# Future Prospects of Spectral Clustering Approaches in Proteomics

**DOI:** 10.1002/pmic.201700454

**Published:** 2018-07-30

**Authors:** Yasset Perez‐Riverol, Juan Antonio Vizcaíno, Johannes Griss

**Affiliations:** ^1^ European Molecular Biology Laboratory European Bioinformatics Institute (EMBL‐EBI) Wellcome Trust Genome Campus Hinxton Cambridge CB10 1SD UK; ^2^ Division of Immunology Allergy and Infectious Diseases Department of Dermatology Medical University of Vienna 1090 Vienna Austria

**Keywords:** algorithms, computational proteomics, mass spectrometry, spectral clustering

## Abstract

In this article, current and future applications of spectral clustering are discussed in the context of mass spectrometry‐based proteomics approaches. First of all, the main algorithms and tools that can currently be used to perform spectral clustering are introduced. In addition, its main applications and their use in current computational proteomics workflows are explained, including the generation of spectral libraries and spectral archives. Finally, possible future directions for spectral clustering, including its potential use to achieve a deeper coverage of the proteome and the discovery of novel post‐translational modifications and single amino acid variants.

Mass spectrometry (MS) based proteomics has become a robust and unique approach to profile the protein composition of complex biological samples. In the most popular data‐dependent acquisition (DDA) approaches, precursor ions are selected according to their abundance, and a number of them (the top *n* ions) are fragmented into MS/MS spectra for further analysis. In contrast, data‐independent acquisition (DIA) approaches implement a parallel fragmentation of all precursor ions, regardless of their intensity or other characteristics, creating a complete digital record of the sample.^1^

The most common method to identify mass spectra in DDA approaches is database searching, where the acquired spectra are compared to generated (theoretical) ones coming from peptide sequences drawn from a given protein sequence database (e.g., UniProt^2^). Database searching has been invaluable in automating the characterization of tandem mass spectra and facilitating proteomics analyses.^3^ However, this methodology still has limitations such as i) spectra remain unidentified due a low signal‐to‐noise ratio of fragment peaks; ii) the underlying peptide is not present in the protein sequence database used; and iii) unanticipated peptide sequences that can change the fragmentation pattern or shift the expected mass of fragment ions, including peptides containing post‐translational modifications (PTMs), artefactual modifications, single amino acid variants (SAAVs), or splicing sites. As a result, on average, approximately 70–75% of analyzed DDA spectra can remain unidentified in an average experiment.^4^, ^5^

Multiple alternative methods have been developed that can increase the proportion of assigned spectra, which can be used alone or in combination: i) the use of multiple sequential sequenced‐based search engines^6^; ii) dependent peptide^7^ and open modification searches^8^; iii) de novo sequencing^9^; and iv) and spectral library searching.^10^ Spectral library searching is the only one of the mentioned methods that at present does not dramatically increase the search time and reuses data already obtained in previous experiments.^10^ Spectral library search engines, such as SpectraST^11^ or BiblioSpec,^12^ use spectral libraries generated from previously identified spectra to match observed MS/MS spectra.^10^ In addition to providing a complementary method to database searches in DDA experiments, spectral library searching has become a central step in DIA approaches, such as SWATH‐MS experiments.^13^ Here, precursors within defined *m*/*z* widows are cofragmented, resulting in complex and convoluted MS/MS spectra. Extracted ion chromatograms (XICs) of the fragments are generated and the coeluting peaks of the fragments of each precursor are used in the quantitative analysis.^14^ In the most‐used methods currently, spectral libraries generated from previous DDA analyses are utilized in the analysis. Ideally, the spectral library should be generated on the same MS instrument used to acquire the SWATH‐MS data, as the correlation of the fragment intensities for a peptide acquired on different instruments has been shown to be potentially low.^15^

Spectral clustering algorithms aim to accurately and efficiently group large numbers of spectra based on their similarity, such that all spectra in a given cluster belong to the same analyte (peptides in this case). The basis of any spectral clustering algorithm relies on three main components: i) assessing the similarity between spectra (distance function); ii) creating clusters of related spectra on the basis of pairwise similarities; and iii) constructing a representative or consensus spectrum for each resulting cluster.^16^ The differences between algorithms and tools depend on how these principles are implemented and which preprocessing steps are used prior to the actual clustering step (e.g., intensity normalization and peak picking).

## 1 Existing Spectral Clustering Algorithms and Their Applications

The first two spectral clustering algorithms tailored for proteomics approaches were MS2Grouper^16^ and Pep‐Miner,^17^ introduced in 2004–2005. The main focus of these tools was to group mass spectra from individual experiments prior to the identification process, in order to decrease the running time (and computation requirements) of database‐based searches. This process achieved a reduction in the number of spectra searched by around 20%, with a reasonable trade‐off of a 1% reduction in the number of peptides identified (in datasets of ≈50 000 spectra).^16^ Nevertheless, this methodology was not adopted into any popular pipeline or search engine. In 2007 Frank et al. introduced the MS‐Cluster algorithm with the same main goal in mind.^18^ The algorithm was able to cluster more than 10 million MS/MS spectra, which led to a tenfold reduction in the amount of data that had to be analyzed. More importantly, they showed that the search results were more accurate when spectral clustering was performed prior to the identification. Additionally, Frank et al. already formulated the idea that spectral clustering could furthermore be used to target unidentified spectra of interest.

In 2007, Lam and cols. introduced the spectral library search engine SpectraST. This tool provides an additional module for spectral clustering and spectra library building, enabling users to build custom spectral libraries. The original algorithm was validated using 1.3 M identified spectra from PeptideAtlas.^11^ SpectraST was extended in 2013 to build spectral libraries from sets of unidentified spectra^19^ and used to study the source of tick blood meals. Most importantly, this was, as far as we are aware, the first time that new biological knowledge was directly derived from clusters of unidentified MS/MS spectra.

The main focus of algorithm development then moved from clustering individual (relatively small) experiments to large data volumes. In 2011, Frank et al. improved MS‐Cluster, and managed to cluster over 500 million spectra simultaneously. In this case, they clustered already analyzed datasets that contained both identified and unidentified spectra. Some of the identified spectra were then clustered with similar unidentified spectra, which enabled the authors to infer additional peptide identifications. This phenomenon was also observed across MS runs coming from different species. Additionally, the authors introduced the concept of spectral archives, which can keep representative consensus spectra of all spectra (including both identified and unidentified ones) and act as a data storage and compression mechanism for large data volumes (including, e.g., public data repositories).

**Figure 1:**
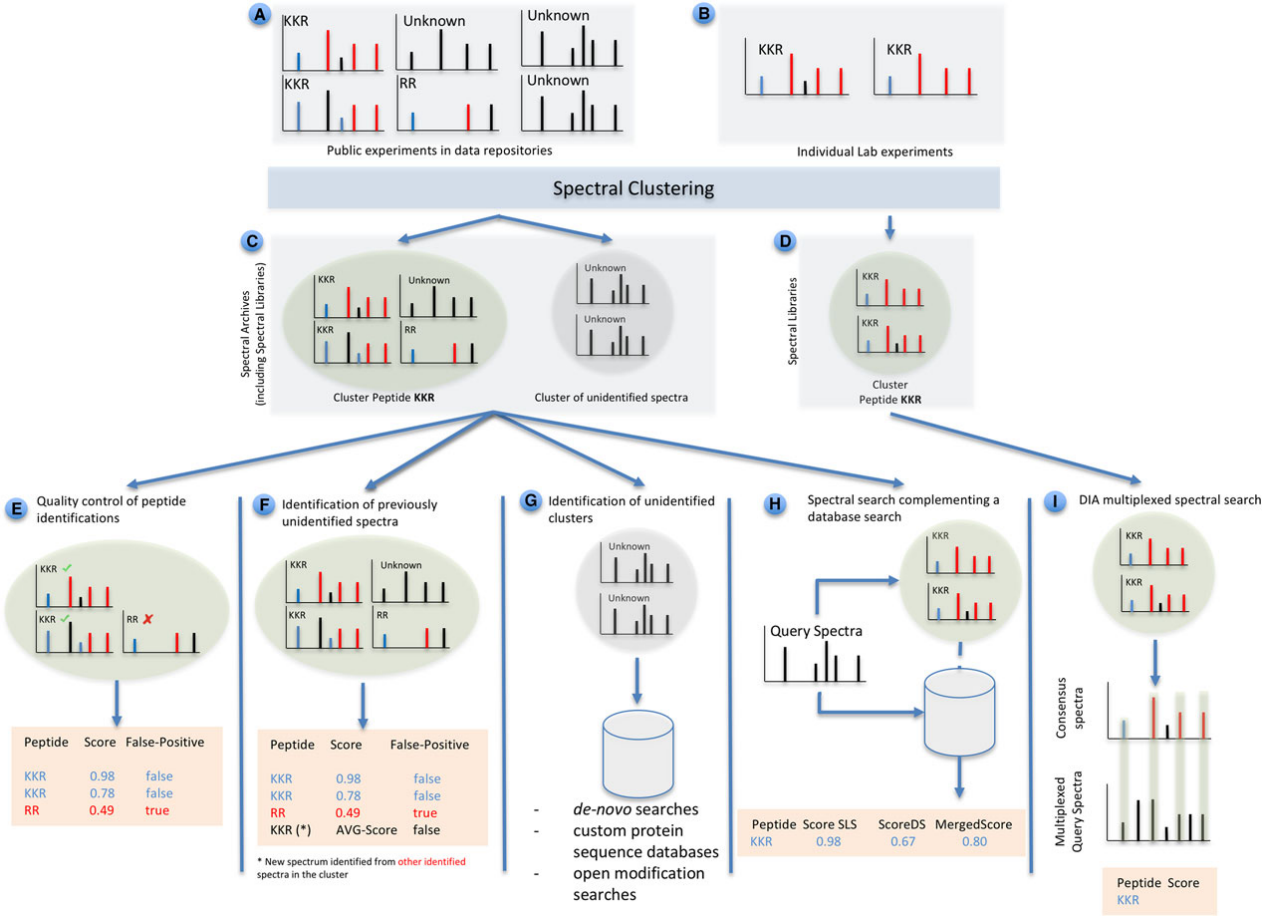
Spectral clustering in proteomics. The input data for any clustering algorithm consists of A) publicly available mass spectra data in proteomics repositories (unidentified, correctly identified, and/or incorrectly identified spectra); B) identified spectra from small‐scale experiments. After the spectral clustering process one main output is expected: C) spectral archives. The spectral archives contain two types of clusters: D) clusters with identified spectra (spectral libraries) and clusters of unidentified spectra. Multiple applications are represented: E) by clustering high‐quality peptide identifications with low‐quality ones, quality assessment of possible false positive identifications can be performed. F) Spectral clustering can help to infer identifications for unidentified spectra, by clustering identified and unidentified spectra together. G) Detection of clusters of unidentified spectra. The resulting clusters should be analyzed with alternative methods such as de novo or open modification searches. H) The combination of database searches with spectral library searches can be useful to increase the number of identifications. I) Finally, spectral libraries in DIA analysis algorithms where spectral assays are designed from previous spectral libraries generated from DDA data.

Two years later, in 2013, we introduced the first version of the PRIDE Cluster algorithm and the corresponding resource.^20^ Based on the concepts formulated by Frank et al., we developed an adapted version of MS‐Cluster, called PRIDE Cluster, which was able to cluster all publicly available identified spectra at the time in the PRIDE database (≈21 million), one of the most prominent public repositories for MS proteomics data^21^ (Figure 1A). The primary goal was to detect and validate correct peptide identifications within the very heterogeneous data stored in PRIDE. This approach followed a simple concept: if the same spectrum (defined as “being in the same spectral cluster”) was identified as the same peptide sequence across different experiments, most likely it was a correct identification. We used validated identifications to automatically create spectral libraries, including species not yet covered by other resources. Validation (quality control) of identifications is considered then as another interesting application of spectral clustering.

In 2016 we extended this approach to cluster all spectra available in PRIDE, including both identified and unidentified, and developed a new spectrum clustering algorithm called *spectra‐cluster*, that made use of Apache Hadoop (http://hadoop.apache.org/), an open source technology commonly used in “big data” analysis. We clustered 256 million spectra and recognized three classes of spectra: i) correctly identified spectra (Figure 1E,F); ii) consistently incorrectly identified spectra (Figure 1E); and iii) reproducibly unidentified spectra (Figure 1G). In a targeted reanalysis, we showed that a significant proportion of the reproducibly unidentified spectra seemed to originate from spectra with unexpected PTMs and/or SAAVs. This highlighted the use of spectral clustering as a tool to achieve a greater depth of the proteome. In fact, the PRIDE Cluster resource (http://www.ebi.ac.uk/pride/cluster/) currently provides access to different compiled sets of commonly observed unidentified spectra, for reanalysis by the community.

Also in 2016, The and Käll introduced the MaRaCluster algorithm.^22^ In contrast to all other approaches, MaRaCluster uses a rarity‐based distance model and complete‐linkage clustering. Thereby, MaRaCluster ignores the actual intensities of fragment ions but focuses on peaks only shared by a few number of spectra for the clustering process. This approach made MaRaCluster less error‐prone to chimeric spectra, a common limitation of these approaches.

## 2 Future Applications of Spectral Clustering

In our opinion, spectral clustering will become more popular mainly for two of the applications outlined above. The first one is the generation of accurate and complete spectral libraries (of identified spectra).^13^ We believe that their use will keep increasing both for DDA, but especially for the increasingly popular DIA approaches. In the case of DDA, the combination of different database search engines has proven to increase the number of identifications between 10 and 20% (see, e.g., ref. 6, 23), in parallel to a huge increase in running time. However, when combining spectral library and database searches (Figure 1H) the compute time does not increase dramatically, providing a higher sensitivity than when two sequence‐based search engines are combined. The combination of both approaches is becoming increasingly popular and has captured the attention of popular tools such as the Trans‐Proteomics Pipeline (TPP)^24^ and MASCOT (Matrix Science, http://www.matrixscience.com/help/spectral_library.html). Furthermore, spectral libraries are essential in the design of spectral assays for the analysis of DIA data (e.g., SWATH‐MS). At the time of writing, at least four tools can be used for the analysis of SWATH‐MS data which are mainly based on spectral libraries: Spectronaut,^25^ OpenSWATH,^26^ Skyline,^27^ and PeakView (SCIEX). All of them enable the construction of spectral libraries using the in‐built clustering algorithms implemented in SpectraST or BiblioSpec.^28^ A study by Navarro et al.^13^ observed a strong overlap of identifications provided by these four spectral library‐based software tools, highlighting the big potential of DIA analysis based on spectral searches for improving reproducibility, for example, in clinical settings.

In our opinion, quality assessment of peptide identifications is the second main application where spectral clustering will play a major role. Recently, different studies highlighted considerable differences in the performance of search engines for peptide–protein identifications.^6^, ^29^ These differences have been extensively observed in the PRIDE Cluster resource (http://www.ebi.ac.uk/pride/cluster/).^4^ Based on the clustering results, we provide sets of validated peptide identifications. Processing repository‐sized datasets is in our opinion a core application of spectral clustering algorithms.

However, probably the most exciting current application of spectral clustering is to recognize reproducibly observed unidentified spectra. This approach can be applied to both small (individual datasets) and large‐scale data volumes (as explained above in the case of PRIDE datasets). These commonly observed unidentified spectra can subsequently be targeted for more in‐depth analysis, by using de novo sequencing or the increasingly popular open modification searches. It is not unreasonable to assume that a substantial proportion of these unidentified spectra corresponds to unknown peptide sequence variants or peptides containing unexpected PTMs. This approach is highly attractive to increase the depth of the coverage of the human proteome, including the detection of novel peptidoforms and proteoforms of biological importance. In this context, we are convinced that spectral clustering can be an essential tool to reuse and derive new biological knowledge from public proteomics datasets.

The original goal of spectral clustering, to reduce the amount of data required to be processed by search engines will, in our view, most likely continue to play a minor role. Nowadays, computational power is not a limiting factor in most approaches. However, it has been shown that the resulting consensus spectra can be of better quality than the best recorded spectrum for a given peptide,^30^ improving the sensitivity of the analysis. In addition, additional PSMs can be inferred by clustering identified with unidentified spectra (Figure 1F). This approach could reduce a major bottleneck of spectral library searching, when users often find existing libraries not suited to their needs, but do not want to invest the often considerable efforts to build their own libraries. We also found that this approach can be used to improve the detectability of low‐abundant proteins and increase the accuracy of label‐free quantification methods (unpublished data).

The efforts to produce massive amounts of spectral data from synthetic peptides will additionally increase the use of spectral clustering for validation purposes.^31^ ProteomeTools (http://www.proteometools.org/), aims to synthesize ≈1.4 million individual peptides to cover all human proteins. The first iteration of the project has already delivered the synthesis and LC–MS/MS analysis of >330 000 synthetic tryptic peptides, covering essentially all canonical human proteins in UniProtKB/Swiss‐Prot. All the MS data has been made publicly available, so researchers are now able to cluster their own experimental data with these spectra, representing “ground‐truth” identifications. Clusters of these synthetic peptides can then be potentially used as gold‐standard identifications and to validate and quality‐control the identification results. These synthetic peptides are a very valuable tool to benchmark the accuracy of spectral clustering algorithms. However, undoubtedly, more research is needed in this particular domain.

## 3 Computational Challenges

Despite highly attractive potential applications, the overall use of spectral clustering algorithms has been so far low. One of the main limitations is the lack of “user‐friendly” software tools to use them. In fact, all algorithms are currently only accessible as command line tools, which makes this technique only available to groups with sound bioinformatics and software development skills. Fortunately, this might soon change through the integration of algorithms into common proteomics software tools. Work is under way to integrate MaRaCluster into OpenMS (https://github.com/OpenMS/, accessed March 30, 2018) and we will soon release a Proteome Discoverer node for the *spectra‐cluster* algorithm. In our view, these two (and related future) developments will considerably increase the accessibility to spectral clustering algorithms.

A second challenge is the lack of a standard file format to exchange MS/MS clustering results. The proteomics community has recently started the development of a such spectral library standard format (https://github.com/HUPO-PSI/SpectralLibraryFormat), which will support the representation of spectral libraries, spectral archives, and intermediate clustering results.^32^ We envision that the development of such standard file format will accelerate the development of new algorithms, tools, and research around spectral clustering.

Two recent studies^33^, ^34^ showed considerable differences in the evaluation of spectral clustering algorithms, with regard to accuracy, and compute performance. There are several unresolved challenges in this area. In fact, the current metrics used to benchmark spectral clustering algorithms, namely cluster homogeneity (purity), cluster completeness (within‐cluster entropy), and peptide completeness (within‐peptide entropy), need to be standardized. More importantly, new gold‐standard datasets have to be generated, annotated, and deposited in public databases to enable unbiased comparisons.

Furthermore, it is important to highlight that spectral clustering represents an attractive platform for the development of “big data” methodologies in proteomics, including the adaptation or extension of existing algorithms to work with large data volumes, for instance in the context of public repositories like PRIDE or MassIVE. During the development of the *spectra‐cluster* algorithm, we explored for the first time the use of “big data” technologies (Hadoop)^35^ to efficiently handle huge data volumes (see above).^4^, ^33^

Finally, we believe that spectral clustering can be a valuable tool in other fields using MS as an analytical platform (for MS/MS based data). For instance, the *spectra‐cluster* algorithm has already been applied to MS/MS lipidomics data.^36^ The same analogous principles and possible applications would be applicable there.

## Conflict of Interest

The authors declare no conflict of interest.
